# Quantitative evaluation of interim positron emission tomography in peripheral T-cell lymphoma

**DOI:** 10.1186/s13550-021-00827-1

**Published:** 2021-09-14

**Authors:** Lars Kurch, Ulrich Dührsen, Andreas Hüttmann, Thomas W. Georgi, Osama Sabri, Regine Kluge, Dirk Hasenclever

**Affiliations:** 1 Klinik Und Poliklinik Für Nuklearmedizin, Universitätsklinikum Leipzig, Leipzig, Germany; 2 Klinik Für Hämatologie, Universitätsklinikum Essen, Essen, Germany; 3 Institut Für Medizinische Informatik, Statistik Und Epidemiologie, Universität Leipzig, Leipzig, Germany

**Keywords:** Peripheral T-cell lymphoma, Positron emission tomography, Interim evaluation, Deauville scale, ∆SUV_max_, qPET

## Abstract

**Background:**

Interim [^18^F]fluoro-deoxyglucose-positron emission tomography predicts outcome in peripheral T-cell lymphoma (PTCL). We compared two quantitative evaluation methods.

**Methods:**

Interim scans from 43 patients with anaplastic lymphoma kinase-negative PTCL from the ‘Positron Emission Tomography-Guided Therapy of Aggressive Non-Hodgkin Lymphomas’ trial were re-analyzed by qPET (relating residual lymphoma-related uptake to liver uptake) and ∆SUV_max_ (relating interim scan to baseline scan). The endpoint was progression-free survival.

**Results:**

qPET and ∆SUV_max_ were closely correlated (Pearson’s *r* = 0.627). Up to the 60^th^ percentile of values ranked by increasing residual activity, the positive predictive value for progression or death increased from 60 to 95%, with stable negative predictive values (NPV) of 60%. Beyond the 60^th^ percentile, the NPV decreased to 40%. qPET ≥ 2 and ∆SUV_max_ < 50% identified high-risk populations comprising 41.9% and 39.5% of patients, with 3-year progression-free survival rates of 5.6% (95% confidence interval, 0.8–37.3) and 0%, respectively, as compared to 63.7% (47.4–85.8) and 61.3% (45.1–83.3) in low-risk patients.

**Conclusions:**

qPET and ∆SUV_max_ identify large fractions of PTCL patients destined to experience treatment failure. qPET may be preferred because it requires a single PET scan, halving the diagnostic effort.

**Supplementary Information:**

The online version contains supplementary material available at 10.1186/s13550-021-00827-1.

## Introduction

Peripheral T-cell lymphoma (PTCL) is a rare disease in Europe and North America, with a yearly incidence of less than 1 in 100,000. It encompasses several entities, which are mainly of aggressive behavior. Response to chemotherapy is generally poor, with long-term survival rates of 30% [1]. Early identification of impending treatment failure may improve outcome.

In a subgroup analysis of the ‘Positron Emission Tomography-Guided Therapy of Aggressive Non-Hodgkin Lymphomas’ (PETAL) trial [2] we showed that interim [^18^F]fluoro-deoxyglucose-positron emission tomography/computed tomography ([^18^F]-FDG-PET/CT) predicts outcome in all major PTCL subtypes except anaplastic lymphoma kinase (ALK)-positive anaplastic large cell lymphoma (ALCL) which has a much better prognosis than the other entities [3]. The ∆SUV_max_ method, comparing the maximum standardized uptake values (SUV_max_) at baseline and interim scanning, appeared better suited than the interim PET-based Deauville 5-point scale with a threshold at score 4 (residual lymphoma-related activity above liver) to predict treatment failure. Score 5 (residual activity markedly above liver) performed better, but its imprecise definition hampers clinical use [4].

To overcome the limitations of visual assessment, we employed qPET (q, quantitative) that relates the SUV of the most intense residual lymphoma-related lesion to the mean SUV of the liver. This procedure, pioneered in pediatric [5] and adult Hodgkin’s lymphoma [6] and confirmed in DLBCL [7], transforms the ordinal Deauville scale into a continuous scale with clearly defined borders between response categories. In this report, we apply qPET to the PTCL population of the PETAL trial and compare it with ∆SUV_max_.

## Patients and methods

### Study design

The PETAL trial (ClinicalTrials.gov NCT00554164; EudraCT 2006-001641-33) was a multicenter study for newly diagnosed aggressive non-Hodgkin lymphomas [2]. The study was approved by the Federal Institute for Drugs and Medical Devices and the ethics committees of all participating sites. All patients gave written informed consent.

PTCL patients were treated with bi-weekly CHOP (cyclophosphamide, doxorubicine, vincristine, prednisone), with a 3-week interval between cycles 2 and 3 to prevent false-positive results at interim staging uniformly performed after cycle 2. Patients with favorable interim [^18^F]-FDG-PET response received four more cycles of CHOP. Patients with unfavorable response were randomly assigned to receive six additional cycles of CHOP or a more intensive protocol [2]. Because the modifications failed to change outcome [2], all treatment arms were combined in the present analysis. High-dose chemotherapy with autologous blood stem cell transplantation was reserved for relapse (*n* = 5), and allogeneic transplantation for second relapse (*n* = 1).

### PET/CT imaging and evaluation

Scanning conditions and image analysis have been described previously [2, 7]. Archived baseline and interim [^18^F]-FDG-PET/CT images were re-analyzed by a single experienced physician (> 5000 evaluated lymphoma scans).

qPET was calculated by dividing the SUV_peak_ of the hottest residual lesion on the interim scan by the SUV_mean_ of the liver. Both parameters were measured semiautomatically using a software tool developed in cooperation with Hermes Medical Solutions AB, Sweden. To determine the SUV_peak_, a volume of interest (VOI) was drawn around the residual. Within this VOI the software automatically identified the maximum SUV voxel and the three hottest adjacent voxels which were used to calculate the mean SUV (SUV_peak_). The SUV_mean_ of physiological liver uptake was measured using a cuboid VOI of 30 ml (edge length proportion length:width:height = 2:2:1) which was placed in the right liver lobe (normally 7th/8th segment) [5]. Based on our previous results [5–7], qPET was used to define quantitative thresholds between the Deauville scores (qDS).

ΔSUV_max_ was determined by dividing the SUV_max_ of the hottest residual lesion on the interim scan by the SUV_max_ of the hottest lesion on the baseline scan [2]. High risk of treatment failure was defined as SUV_max_ reduction < 50% [3].

### Statistical analysis

The study endpoint was progression-free survival (PFS) defined as time from interim [^18^F]-FDG-PET scanning to disease progression, relapse, or death from any cause. For simplicity, we treated PFS as a binary variable (i. e. whether or not events occurred within 60 month). This appeared justified, because, in PTCL, the majority of events occur within the first two years [8], and, with a median follow-up of 52 months, the data were mature [2, 3].

We plotted empirical cumulative distribution functions (ECDFs) of qPET and used the area under the Receiver Operating Characteristic (ROC) curve to compare qPET and ∆SUV_max_. Instead of ∆SUV_max_, we used 1-ΔSUV_max_, i.e. the remaining proportion of [^18^F]-FDG uptake, which allowed us to use the log scale (no negative values) and rendered the correlation with qPET positive [7]. All analyses were carried out in R, version 3.5.1 (R Core Team, Vienna, Austria).

## Results

### Patients characteristics

Of 862 patients treated in the PETAL trial, 76 had PTCL [2]. Baseline and interim scans for post-hoc analyses were available from 57 patients (Additional file 1: Fig. S1). Baseline characteristics of included and excluded patients were similar. Forty-three patients had one of several types of ALK-negative PTCL, and 14 had ALK-positive ALCL (Table 1).

**Table 1:** Baseline characteristics of T-cell lymphoma patients

	ALK-neg. peripheral T-cell lymphomas		ALK-pos. anaplastic large cell lymphoma	
	Included	Excluded	Included	Excluded
No. of patients	43	12	14	7
Peripheral T-cell lymphoma, not otherwise specified	15	5	n.a	n.a
Angioimmunoblastic T-cell lymphoma	13	5	n.a	n.a
ALK-negative anaplastic large cell lymphoma	12	1	n.a	n.a
Unclassified T-cell lymphoma	3	1	n.a	n.a
Age – median (range), years	61 (26–77)	70 (29–79)	34 (20–66)	32 (19–54)
Male sex	30 (70%)	9 (75%)	9 (64%)	7 (100%)
ECOG performance status ≥ 2	3 (7%)	1 (8%)	0 (0%)	1 (14%)
Ann Arbor stage III or IV	30 (70%)	8 (67%)	5 (36%)	5 (71%)
Extranodal sites > 1	10 (23%)	1 (8%)	2 (14%)	0 (0%)
Lactate dehydrogenase > ULN	25 (58%)	7 (58%)	3 (21%)	1 (14%)
*International Prognostic Index*				
Low risk	16 (37%)	2 (17%)	11 (79%)	5 (71%)
Low-intermediate risk	9 (21%)	6 (50%)	3 (21%)	2 (29%)
High-intermediate risk	12 (28%)	3 (25%)	0 (0%)	0 (0%)
High risk	6 (14%)	1 (8%)	0 (0%)	0 (0%)

ECOG, Eastern Cooperative Oncology Group; ULN, upper limit of normal

### Response assessment

**Fig. 1:**
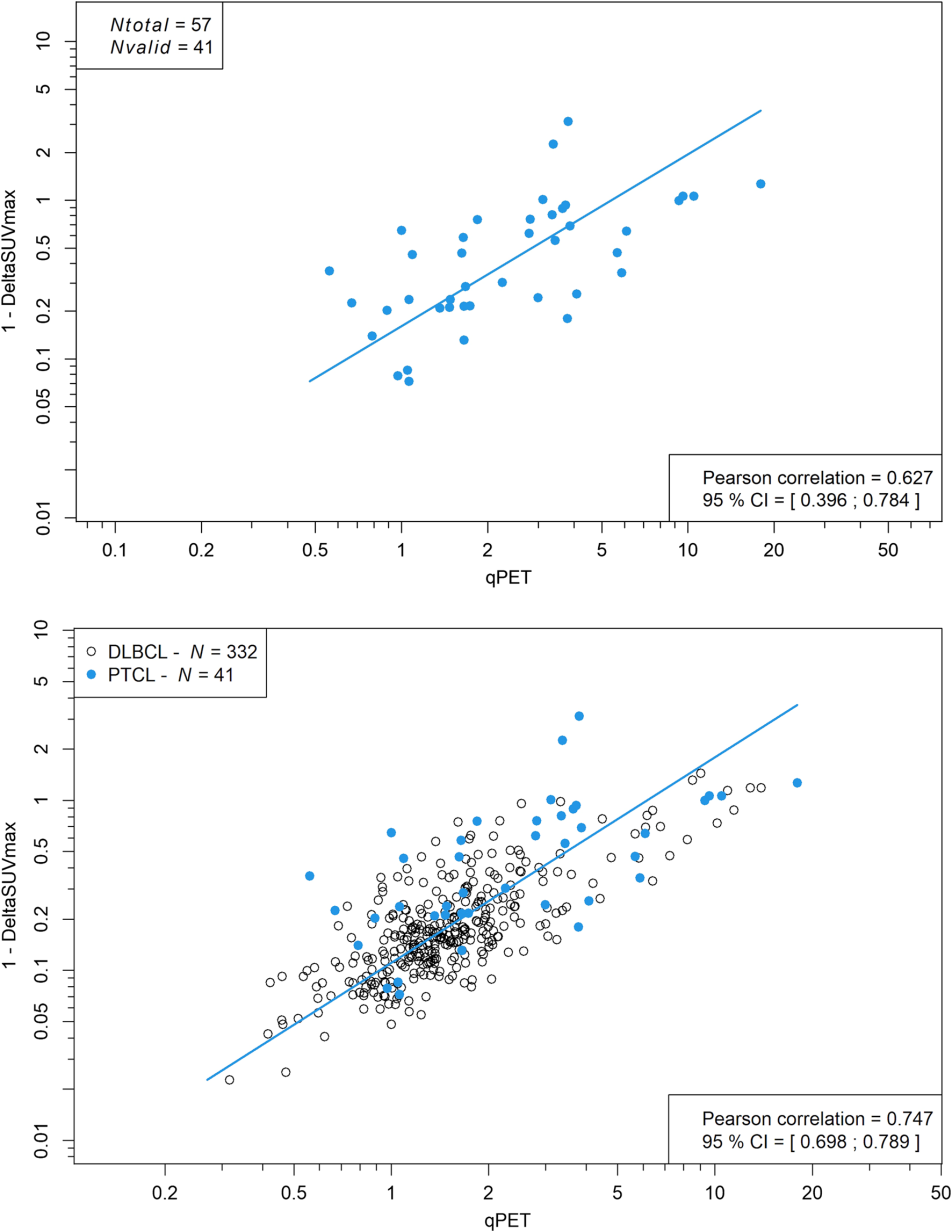
Scatterplot of qPET and ΔSUV_max_ values in peripheral T-cell lymphoma alone (top) and peripheral T-cell-lymphoma (filled blue circles) and diffuse large B-cell lymphoma (open black circles) combined (bottom). The data for diffuse large B-cell lymphoma have been published before [7]. CI, confidence interval; Nvalid, measurable residual activity

**Fig. 2:**
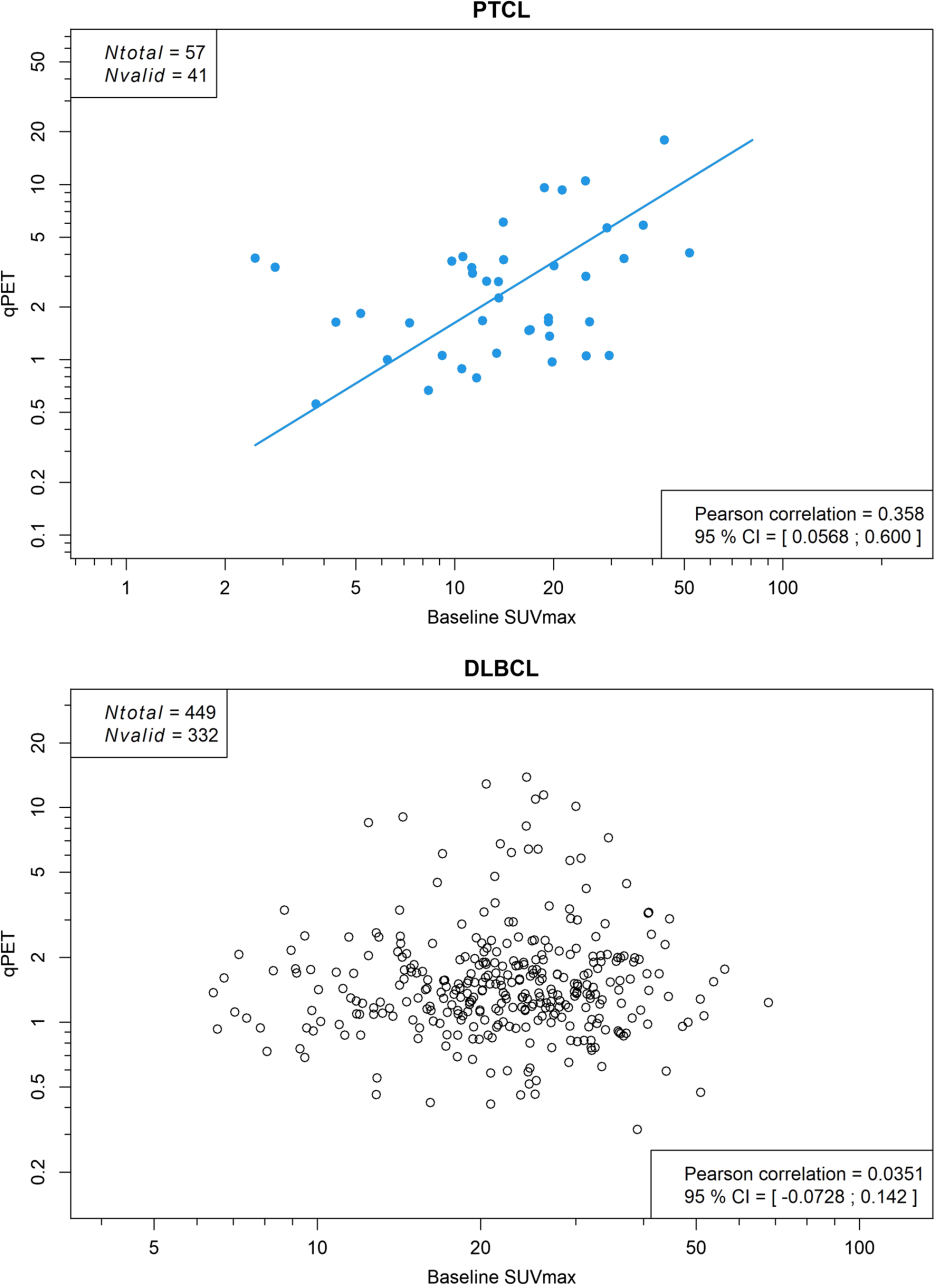
Scatterplot of qPET and baseline SUV_max_ values in peripheral T-cell lymphoma (top) and diffuse large B-cell lymphoma (bottom). CI, confidence interval; Nvalid, measurable residual activity

The ECDFs of SUV findings at interim scanning did not significantly differ between individual entities (Additional file 1: Fig. S2). At interim scanning, residual lymphoma-related activity was measurable in 32 ALK-negative PTCL and nine ALK-positive ALCL. The remaining patients’ scans were devoid of unphysiological activity. qPET and ∆SUV_max_ measurements were closely correlated (Pearson’s *r* = 0.627; 95% confidence interval [CI] 0.396–0.784) and in line with the observations made in DLBCL (Fig. 1). In PTCL, interim qPET showed a moderate correlation with baseline SUV_max_ (Pearson’s *r* = 0.358; 95% CI 0.057–0.600). No such correlation was seen in DLBCL (Fig. 2).

### Outcome prediction

PFS was statistically significantly associated with PTCL subtype, with 3-year PFS rates of 38.9% in ALK-negative PTCL and 85.1% in ALK-positive ALCL (Additional file 1: Fig. S3 ). Since interim PET had failed to predict outcome of ALK-positive ALCL in our previous study [3], we restricted the comparison of qPET and ∆SUV_max_ to ALK-negative PTCL. As judged by ROC analysis, both methods predicted PFS equally well, with almost identical areas under the curve (qPET, 0.775, 95% CI 0.631–0.92; ∆SUV_max_, 0.792, 95% CI 0.66–0.923; *p* = 0.731).

**Fig. 3:**
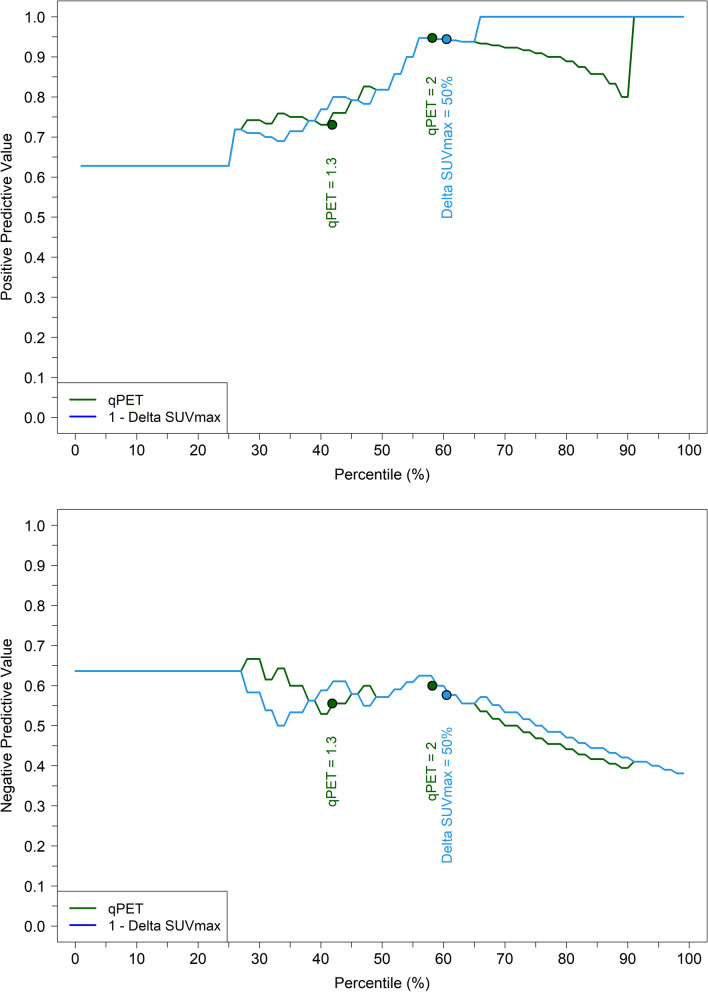
Positive predictive value (top) and negative predictive value (bottom) of corresponding percentiles of qPET and ∆SUV_max_ measurements. The constant part of the curves at low percentiles is due to the inclusion of non-measurable values set at zero (*n* = 16)

To compare the positive and negative predictive values of qPET and ∆SUV_max_ at comparable thresholds, the values were plotted along their respective percentiles. This resulted in largely superimposable curves (Fig. 3). Up to the 60th percentile, the positive predictive value continuously increased from 60 to 95%, while the negative predictive value remained stable at 60%. Beyond the 60^th^ percentile, the negative predictive value decreased to 40%. Figure 3 also shows the positions of the qPET thresholds of 1.3 discriminating qDS3 and qDS4, and 2.0 discriminating qDS4 and qDS5 [5–7], and the 50% ∆SUV_max_ threshold splitting the PTCL population in two prognostic groups [3].

### Outcome according to prognostic group

**Fig. 4:**
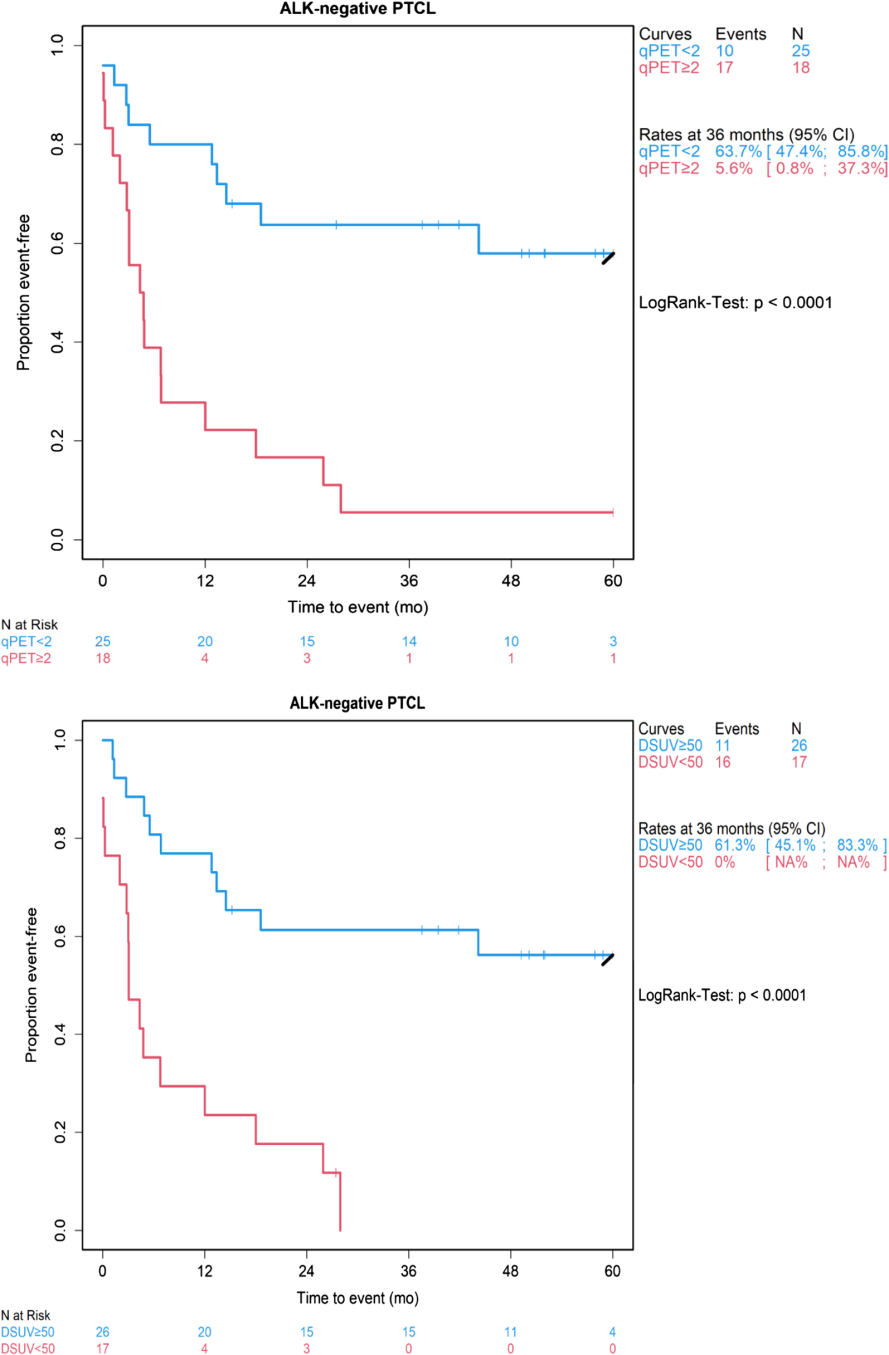
Progression-free survival in prognostic subgroups derived from the quantitative Deauville scale (top) and the ∆SUV_max_ scale (bottom) (Kaplan–Meier analysis). qPET < 2 corresponds to quantitative Deauville score 1–4 (qDS1-4) and qPET ≥ 2 corresponds to qDS5. CI, confidence interval

qDS5 (qPET ≥ 2) identified a high-risk group comprising 18 patients (41.9%), with a 3-year PFS rate of 5.6% (95% confidence interval, 0.8–37.3). By contrast, qDS1-4 was associated with a 3-year PFS rate of 63.7% (47.4–85.8), with similar rates (71.6% and 57.1%, respectively) for qDS1 (qPET = 0; *n* = 11) and qDS2-4 (qPET > 0 to < 2; *n* = 14). An SUV_max_ reduction < 50% was seen in 17 patients (39.5%), with a 3-year PFS rate of 0%, compared to 61.3% (45.1–83.3) in patients with an SUV_max_ reduction ≥ 50% (Fig. 4). Similar results were obtained for overall survival (Additional file 1: Fig. S4).

Multivariable analysis including interim [^18^F]-FDG-PET response and International Prognostic Index (IPI) risk group was hampered by small patient numbers. Irrespective of IPI risk, poor interim [^18^F]-FDG-PET response appeared to be associated with dismal outcome. Whether IPI had an impact on outcome of patients with good interim [^18^F]-FDG-PET response, remained questionable (Additional file 1: Fig. S5).

## Discussion

In the present study, interim [^18^F]-FDG-PET evaluation by qPET and ∆SUV_max_ yielded closely related measurements. Outcome prediction was similar, suggesting that the two methods convey comparable information. Importantly and in contrast to the visual Deauville scale, qPET clearly defines DS5, which is crucial for the identification of PTCL patients at high risk of treatment failure.

The correlation between qPET and ∆SUV_max_ was similar to the findings in DLBCL [7]. qPET was correlated with baseline SUV_max_ in PTCL, but not in DLBCL, reflecting differences in the responsiveness to chemotherapy. In most DLBCL patients, response is rapid and accompanied by a marked reduction in [^18^F]-FDG uptake. In PTCL, chemotherapy is much less effective. Persistently high [^18^F]-FDG uptake translates into high qPET values.

The large sample size (449–898 patients) of our previous qPET studies allowed us to define precise borders between the five Deauville categories [5–7]. Irrespective of disease, the borders identified were identical. Because the relationship between the uptake in a residual lesion and the reference region, as perceived by the reader, should not be disease-specific, we adopted the previously defined quantitative Deauville scale also for PTCL. The small size of our PTCL study precluded a formal confirmation of the thresholds.

Interim assessment in ALK-negative PTCL was characterized by higher positive and lower negative predictive values than in DLBCL [7], reflecting differences in the frequency of treatment failure [2]. qDS5 identified 18 of 43 patients (41.9%) to be at risk of treatment failure, all but one of whom progressed or died within 30 months. An SUV_max_ reduction < 50% allocated 17 patients (39.5%) to the high-risk group, with similarly poor outcome. Both quantitative methods appear suitable to select patients for an early treatment change. qPET may be preferable, because it does not require a baseline [^18^F]-FDG-PET/CT, thus minimizing the influence of factors interfering with the evaluation [7]. However, it is important to mention that the qPET method as described here was developed 10 years ago, based on scanner systems available at that time. With the new PET scanner generations voxel size became markedly smaller so that an adapted qPET calculation is recommendable which is independent of voxel size. In the EuroNet-PHL-C2 and the GPOH-HD2020 registry trials, the SUV_peak_ is calculated as the SUV_mean_ of the hottest connected voxels forming a volume of 0.2 ml (instead of the SUV_mean_ of the 4 hottest voxels) [9].

For patients failing conventional chemotherapy, the most promising treatment is allogeneic transplantation. In a recent first-line trial, transplantation after five treatment cycles proved impossible in almost a third of patients, mainly because of progression beyond cycle 2 [10]. The window for allogeneic transplantation may be narrow in high-risk PTCL. Interim [^18^F]-FDG-PET could help detect impending progression before it is too late.

The major limitation of our study is its small size, inherent in all investigations in rare diseases. The results should be confirmed in an independent cohort, preferably of larger size.

In conclusion, qPET requiring a single [^18^F]-FDG-PET scan identifies similarly large fractions of PTCL patients at risk of treatment failure as does ∆SUV_max_ relying on a comparison of two scans. qPET stringently defines DS5 which is crucial for risk allocation.

## Supplementary Information

**Additional file 1. Supplemental Figure 1**. Patient selection according to the availability and evaluability of metabolic imaging data. Preconditions for study inclusion were the availability of [^18^F]-FDG-PET images at staging and following two courses of CHOP chemotherapy on the central data server, including the opportunity to reliably perform quantitative measurements, i. e. △SUV_max_ and qPET calculations. **Supplemental Figure 2.** Empirical cumulative distribution functions of qPET (top) and △SUV_max_ values (bottom) from interim positron emission tomography for different peripheral T-cell lymphomas. PTCL, peripheral T-cell lymphoma; NOS, not otherwise specified; AITL, angioimmunoblastic T-cell lymphoma; ALK+ ALCL, anaplastic lymphoma kinase (ALK)-positive anaplastic large cell lymphoma; ALK- ALCL, ALK-negative ALCL.** Supplemental Figure 3**. Progression-free survival in anaplastic lymphoma kinase (ALK)-negative peripheral T-cell lymphomas (ALK- PTCL), ALK-positive anaplastic large cell lymphoma (ALK+ ALCL), and diffuse large B-cell lymphoma (DLBCL). The DLBCL data have been published before [7]. CI, confidence interval.** Supplemental Figure 4**. Overall survival in prognostic subgroups derived from the quantitative Deauville scale (top) and the △SUV_max_ scale (bottom) (Kaplan-Meier analysis). qPET<2 corresponds to quantitative Deauville score 1-4 (qDS1-4) and qPET≥2 corresponds to qDS5. CI, confidence interval.** Supplemental Figure 5.** Progression-free survival in prognostic subgroups derived from the International Prognostic Index (score 0-2 versus 3-5) combined with the interim positron emission tomography response as assessed by the quantitative Deauville scale (top) or the DSUV_max_ scale (bottom) (Kaplan-Meier analysis).

## Data Availability

The datasets generated and analyzed are available from the corresponding author upon request.
