# Supplementary material for: Quantitative evaluation of interim positron emission tomography in peripheral T-cell lymphoma
Source: EJNMMI Res. 2021 Sep 14;11:90. doi: 10.1186/s13550-021-00827-1 (PMC8440745; doi:10.1186/s13550-021-00827-1)
Supplement: Supplementary file 1 — Additional file 1. Supplemental Figure 1. Patient selection according to the availability and evaluability of metabolic imaging data. Preconditions for study inclusion were the availability of [18F]-FDG-PET images at staging and following two courses of CHOP chemotherapy on the central data server, including the opportunity to reliably perform quantitative measurements, i. e. △SUVmax and qPET calculations. Supplemental Figure 2. Empirical cumulative distribution functions of qPET (top) and △SUVmax values (bottom) from interim positron emission tomography for different peripheral T-cell lymphomas. PTCL, peripheral T-cell lymphoma; NOS, not otherwise specified; AITL, angioimmunoblastic T-cell lymphoma; ALK+ ALCL, anaplastic lymphoma kinase (ALK)-positive anaplastic large cell lymphoma; ALK- ALCL, ALK-negative ALCL. Supplemental Figure 3. Progression-free survival in anaplastic lymphoma kinase (ALK)-negative peripheral T-cell lymphomas (ALK- PTCL), ALK-positive anaplastic large cell lymphoma (ALK+ ALCL), and diffuse large B-cell lymphoma (DLBCL). The DLBCL data have been published before [7]. CI, confidence interval. Supplemental Figure 4. Overall survival in prognostic subgroups derived from the quantitative Deauville scale (top) and the △SUVmax scale (bottom) (Kaplan-Meier analysis). qPET<2 corresponds to quantitative Deauville score 1-4 (qDS1-4) and qPET≥2 corresponds to qDS5. CI, confidence interval. Supplemental Figure 5. Progression-free survival in prognostic subgroups derived from the International Prognostic Index (score 0-2 versus 3-5) combined with the interim positron emission tomography response as assessed by the quantitative Deauville scale (top) or the DSUVmax scale (bottom) (Kaplan-Meier analysis). [file 13550_2021_827_MOESM1_ESM.docx]

**Data Supplement**

**Quantiative evaluation of interim positron emission tomography**

**in peripheral T-cell lymphoma**

Lars Kurch, Ulrich Dührsen, Andreas Hüttmann, Thomas W. Georgi,

Osama Sabri, Regine Kluge, and Dirk Hasenclever

**Supplemental Figures 1 - 5**

**Supplemental Figure 1**

**
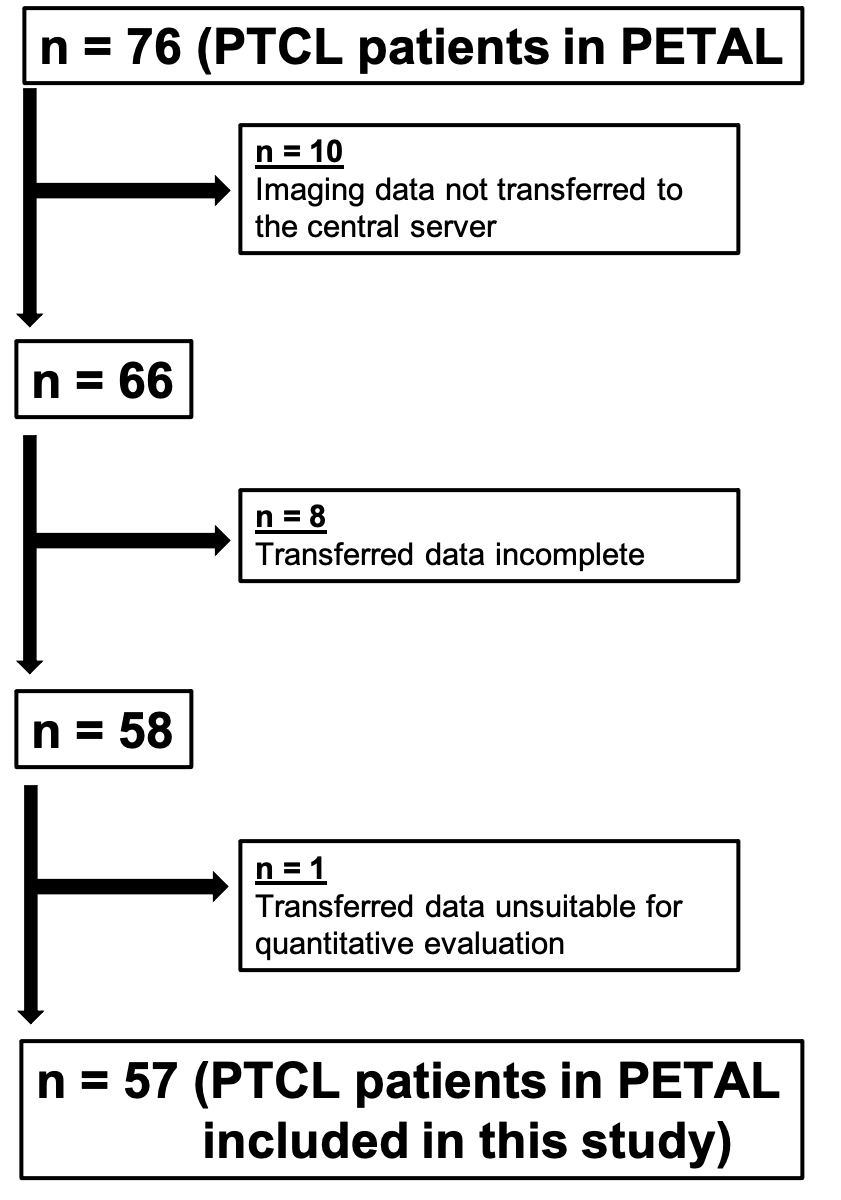
**

Patient selection according to the availability and evaluability of metabolic imaging data. Preconditions for study inclusion were the availability of [^18^F]-FDG-PET images at staging and following two courses of CHOP chemotherapy on the central data server, including the opportunity to reliably perform quantitative measurements, i. e. ΔSUV_max_ and qPET calculations.

**Supplemental Figure 2**


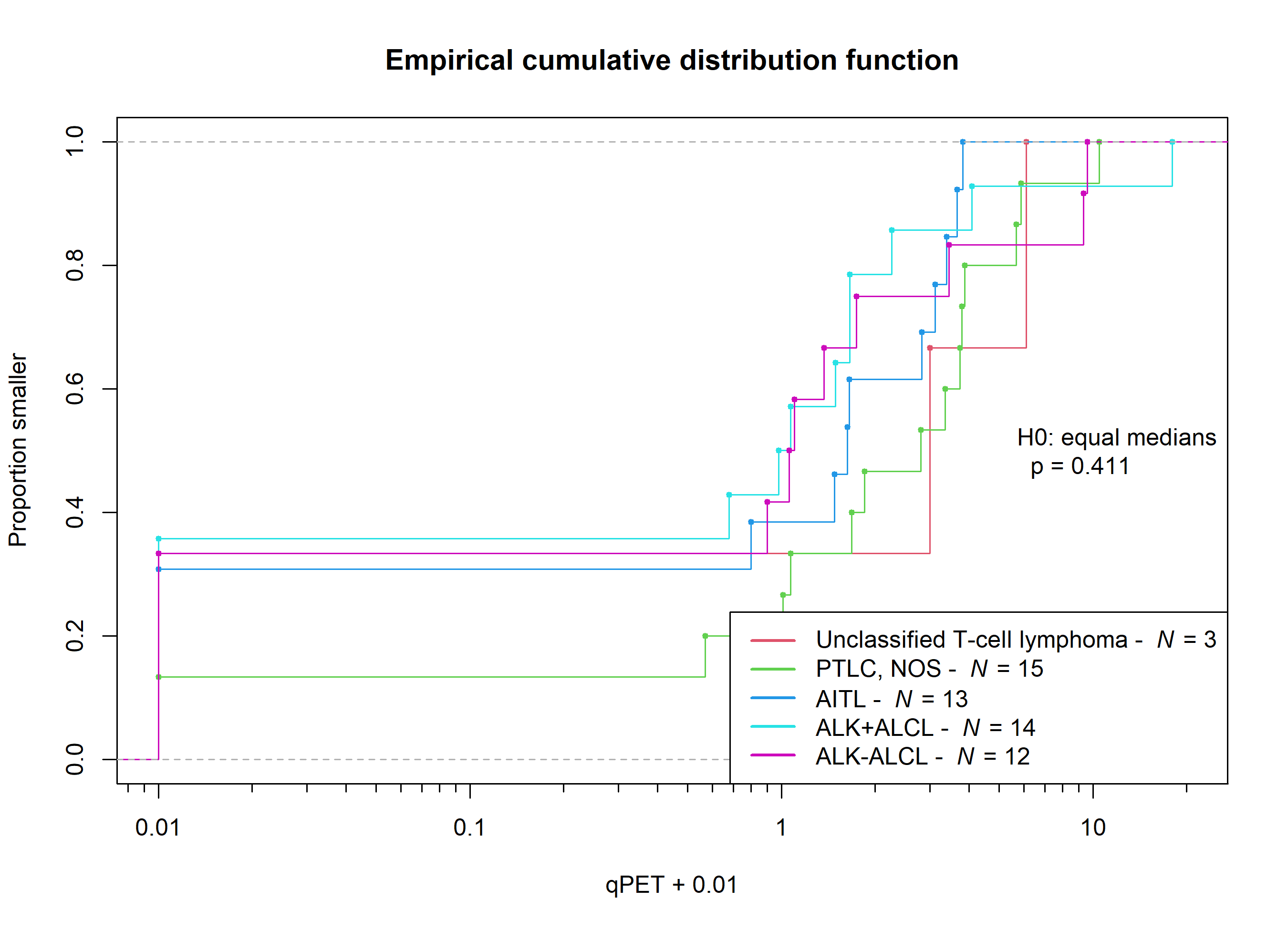

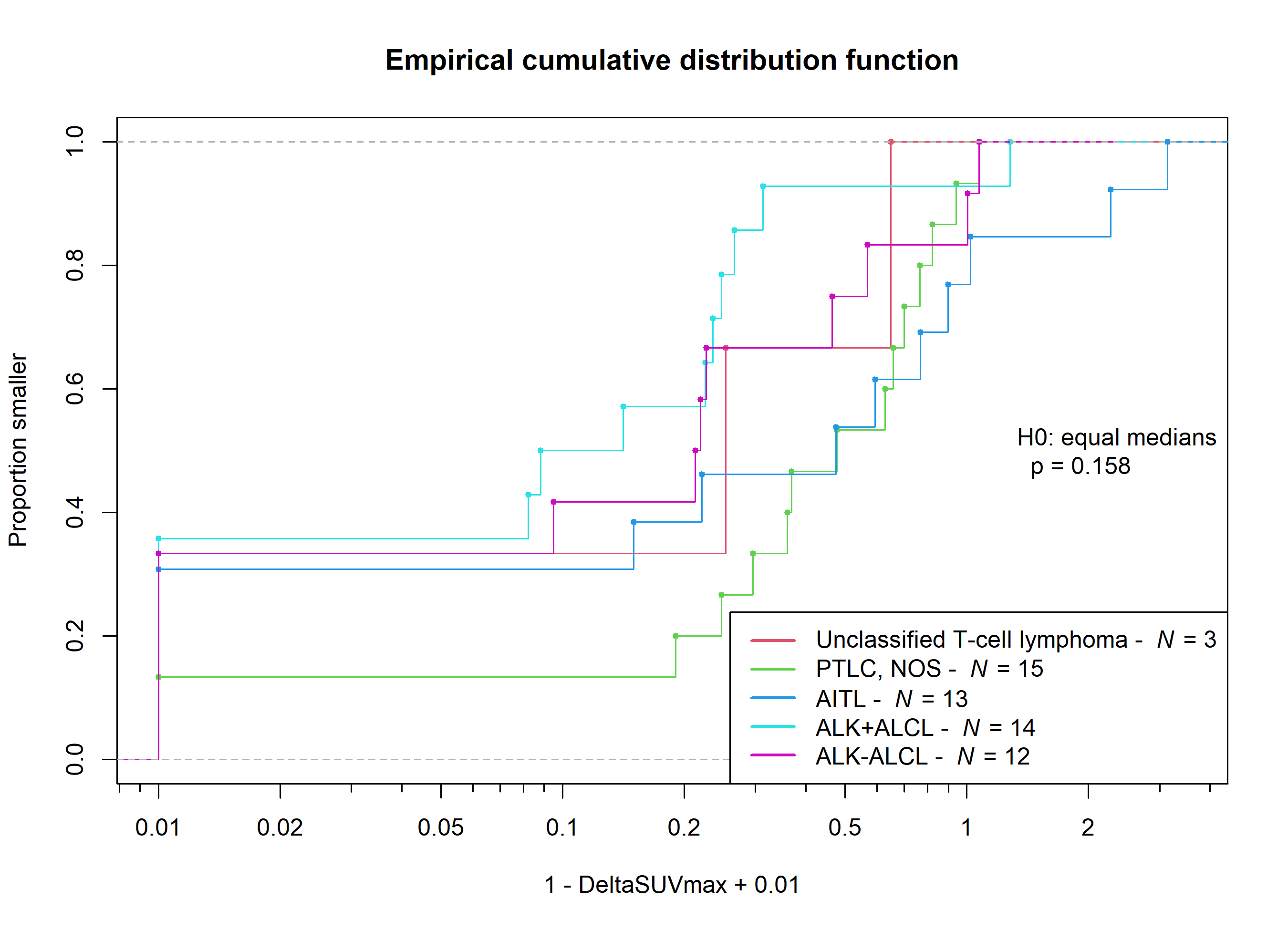


Empirical cumulative distribution functions of qPET (top) and ΔSUV_max_ values (bottom) from interim positron emission tomography for different peripheral T-cell lymphomas. PTCL, peripheral T-cell lymphoma; NOS, not otherwise specified; AITL, angioimmunoblastic T-cell lymphoma; ALK+ ALCL, anaplastic lymphoma kinase (ALK)-positive anaplastic large cell lymphoma; ALK- ALCL, ALK-negative ALCL.

**Supplemental Figure 3**


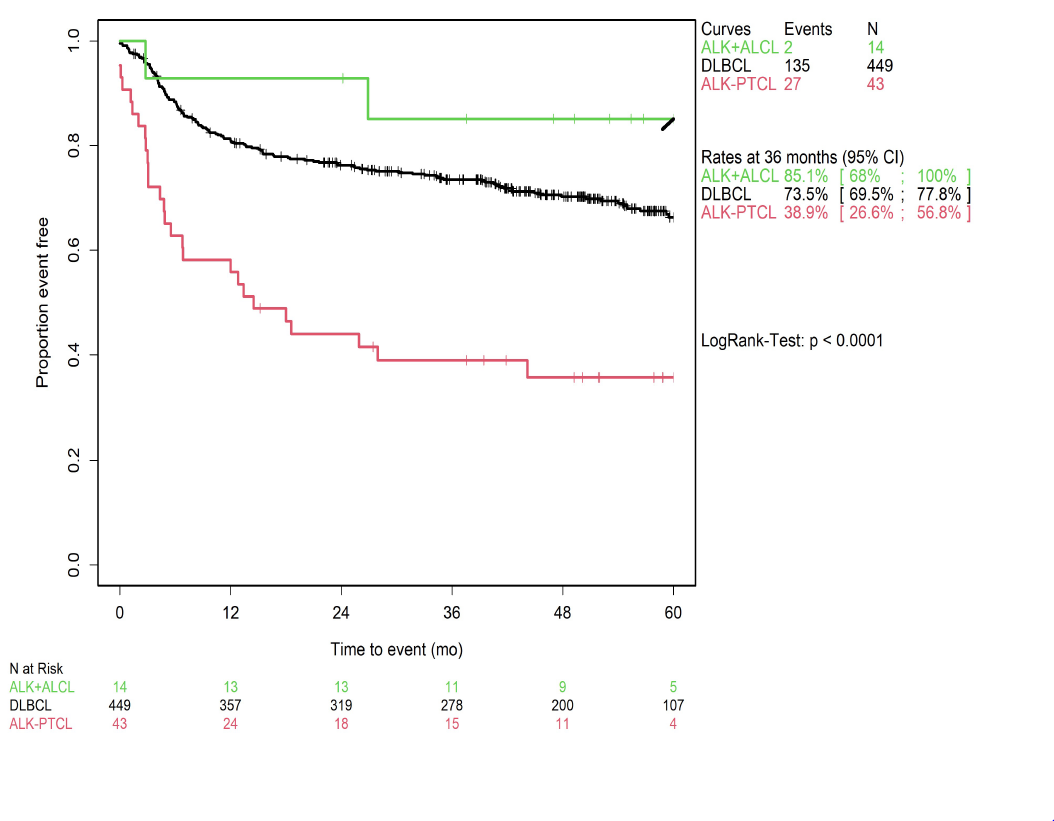


Progression-free survival in anaplastic lymphoma kinase (ALK)-negative peripheral T-cell lymphomas (ALK- PTCL), ALK-positive anaplastic large cell lymphoma (ALK+ ALCL), and diffuse large B-cell lymphoma (DLBCL). The DLBCL data have been published before [7]. CI, confidence interval.

**Supplemental Figure 4**


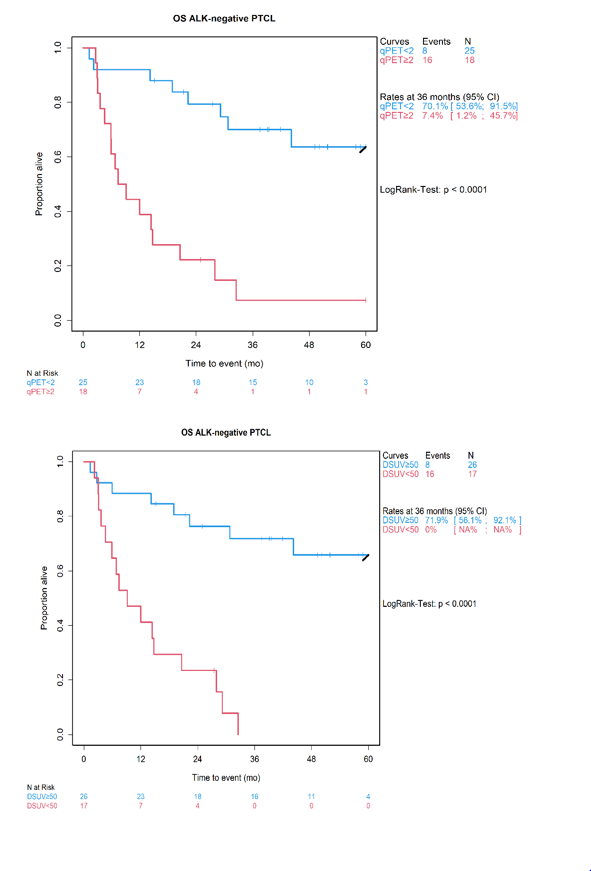


Overall survival in prognostic subgroups derived from the quantitative Deauville scale (top) and the ΔSUV_max_ scale (bottom) (Kaplan-Meier analysis). qPET<2 corresponds to quantitative Deauville score 1-4 (qDS1-4) and qPET≥2 corresponds to qDS5. CI, confidence interval.

**Supplemental Figure 5**


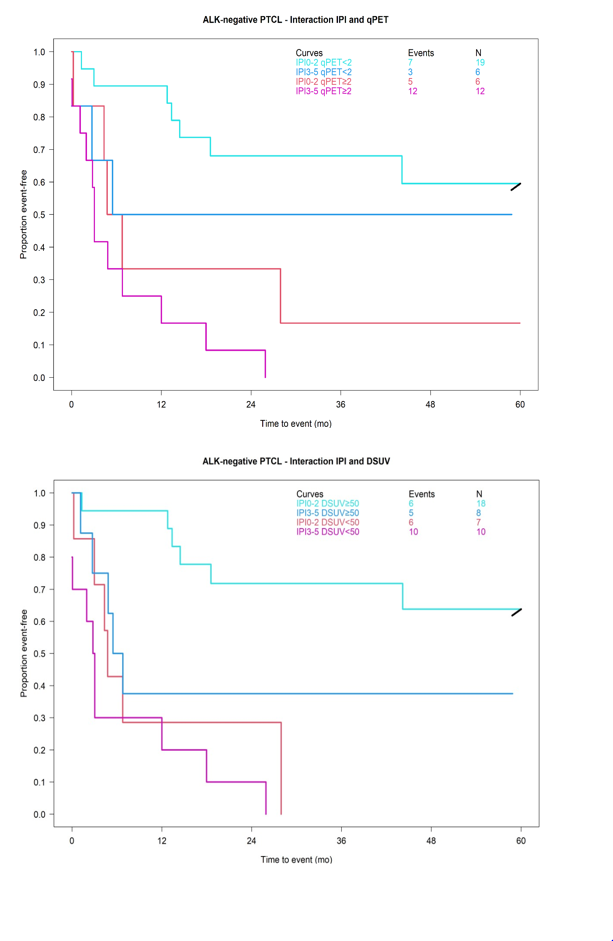


Progression-free survival in prognostic subgroups derived from the International Prognostic Index (score 0-2 versus 3-5) combined with the interim positron emission tomography response as assessed by the quantitative Deauville scale (top) or the DSUV_max_ scale (bottom) (Kaplan-Meier analysis). qPET≥2
